# Serum β-hCG Combined with Traditional Tumor Markers Improves Detection Efficacy and Prognostic Prediction in Cholangiocarcinoma

**DOI:** 10.3390/ijms27125438

**Published:** 2026-06-16

**Authors:** Suppakrit Kongsintaweesuk, Thatsanapong Pongking, Keerapach Tunbenjasiri, Pakornkiat Tanasuka, Sittiruk Roytrakul, Sudarat Onsurathum, Chawalit Pairojkul, Kitti Intuyod, Vor Luvira, Somchai Pinlaor, David Blair, Porntip Pinlaor

**Affiliations:** 1 Biomedical Science Program, Graduate School, Khon Kaen University, Khon Kaen 40002, Thailand; suppa.krit@kkumail.com (S.K.); keerapach_tu@kkumail.com (K.T.); 2 Centre for Research and Development of Medical Diagnostic Laboratories, Faculty of Associated Medical Sciences, Khon Kaen University, Khon Kaen 40002, Thailand; 3 Department of Parasitology, Faculty of Medicine, Khon Kaen University, Khon Kaen 40002, Thailand; thatpo@kku.ac.th (T.P.); psomec@kku.ac.th (S.P.); 4 Department of Pathology, Faculty of Medicine, Khon Kaen University, Khon Kaen 40002, Thailand; pakornkiat.t@kkumail.com (P.T.); chawpa@kku.ac.th (C.P.); kittin@kku.ac.th (K.I.); 5 National Center for Genetic Engineering and Biotechnology, National Science and Technology Development Agency, Pathum Thani 12120, Thailand; sittiruk@biotec.or.th; 6 Department of Microbiology and Parasitology, Faculty of Medical Science, Naresuan University, Phitsanulok 65000, Thailand; sudarato@nu.ac.th; 7 Department of Surgery, Faculty of Medicine, Khon Kaen University, Khon Kaen 40002, Thailand; vor@kku.ac.th; 8 College of Science and Engineering, James Cook University, Townsville, QLD 4811, Australia; davidblair49@gmail.com; 9 School of Medical Technology, Faculty of Associated Medical Sciences, Khon Kaen University, Khon Kaen 40002, Thailand

**Keywords:** bile duct cancer, biomarker, liver cancer

## Abstract

Cholangiocarcinoma (CCA) in Northeast Thailand is characterized by late diagnosis and poor prognosis, creating a critical need for effective early-detection biomarkers. This study utilized a multi-omics approach to identify novel diagnostic targets and improve CCA screening. Initial serum proteomic and transcriptomic analyses revealed significant upregulation of the luteinizing hormone/choriogonadotropin receptor (LHCGR) in CCA patients, correlating with advanced disease stages. Interaction network analysis subsequently identified its circulating ligand, beta-human chorionic gonadotropin (β-hCG), as a highly translatable clinical target. The protein expression of β-hCG was assessed via immunohistochemistry (IHC) in 100 tissue samples, and serum levels of β-hCG, alongside routine markers (CA19-9, AFP, and CEA), were quantified in a cohort of 405 individuals, including 153 CCA patients. IHC confirmed significantly higher β-hCG expression in tumor tissues compared to adjacent areas (*p* < 0.0001). Serum β-hCG levels were significantly elevated in CCA patients and correlated with tumor volume and reduced overall survival. Diagnostically, a combined multiparameter panel (β-hCG, carbohydrate antigen 19-9, carcinoembryonic antigen, and alpha-fetoprotein) yielded excellent accuracy in distinguishing CCA from healthy controls (AUC: 0.962) and hepatocellular carcinoma cases (AUC: 0.890). However, discriminatory efficiency was notably lower when differentiating CCA from benign biliary diseases (AUC: 0.680) and liver metastases (AUC: 0.705). In conclusion, activation of the LHCGR signaling axis is a novel pathophysiological feature in CCA. When integrated into a multi-marker blood panel, circulating β-hCG serves as a valuable complementary risk-stratification and prognostic tool, though further optimization is required to overcome limited specificity in the presence of confounding liver pathologies before routine clinical implementation.

## 1. Introduction

Cancer is among the leading causes of death worldwide, with incidence rates increasing in recent years [1]. Cholangiocarcinoma (CCA), a malignancy of the biliary system, is a significant health burden in the Greater Mekong subregion, where its high prevalence is driven by the parasitic liver fluke, *Opisthorchis viverrini* (OV). The International Agency for Research on Cancer (IARC) classifies this parasite as a Group 1 carcinogen, confirming its definitive role in causing cancer in humans, particularly in Thailand, Lao PDR, and Vietnam [2]. Recent reports indicate that the incidence of CCA in Northeast Thailand is 96 per 100,000 men [3], the highest in the world [4]. Unfortunately, most CCA patients are diagnosed at an advanced stage [5], resulting in a poor prognosis, with a five-year survival rate of less than 5% [6]. Early detection is therefore paramount for improving patient outcomes and facilitating timely intervention [7]. Given CCA’s significant mortality rate and the challenges associated with developing efficient screening or diagnostic methods, it is crucial to identify effective and efficient screening strategies. Currently, several approaches are employed to detect CCA, including ultrasonography, CT scanning and tissue biopsy [8]. However, these methods are costly and not feasible for use in rural areas. Therefore, the identification of accessible, highly sensitive, and specific circulating biomarkers is essential.

The search for effective CCA screening tools has driven intensive research into molecules capable of differentiating malignant tumors from benign biliary diseases (BBD). Established oncological biomarkers, including alpha-fetoprotein (AFP), carcinoembryonic antigen (CEA), and carbohydrate antigen 19–9 (CA19-9), are routinely utilized in clinical practice [9]; however, their limited sensitivity and specificity for CCA necessitate the development of novel diagnostic panels [10]. Among emerging candidates, the glycoprotein beta-human chorionic gonadotropin (β-hCG) has garnered increasing attention [11]. While traditionally recognized for its regulatory role in pregnancy and trophoblastic diseases, β-hCG also exhibits diverse oncogenic functions, including the promotion of tumor progression [12,13]. Ectopic expression of β-hCG has been reported in several gastrointestinal cancers [14,15,16,17], where it often correlates with aggressive tumor behavior and poor clinical outcomes [16,17]. Notably, elevated biliary β-hCG has shown promise as a predictive marker of biliary neoplasia in patients with primary sclerosing cholangitis, a known risk factor for CCA [18]. Despite these intriguing associations, the comprehensive diagnostic and prognostic utility of systemic β-hCG specifically in CCA remains under-investigated.

To uncover novel systemic molecular signatures associated with CCA, modern multi-omics approaches offer a powerful strategy. Integrating serum proteomics with public transcriptomic databases allows for the identification of dysregulated signaling networks that reflect malignancy-related changes more comprehensively than single-analyte approaches. Specifically, investigating the expression profiles of cell-surface receptors, such as the luteinizing hormone/choriogonadotropin receptor (LHCGR), and mapping their corresponding protein–protein interaction networks can identify highly specific circulating ligands. This systems biology approach facilitates the discovery of translatable biomarkers that are secreted directly into the bloodstream, making them ideal targets for routine clinical diagnostics.

In this study, we employed a discovery workflow to identify activation of the LHCGR signaling axis in CCA, prompting the investigation of its circulating ligand, β-hCG, as a novel screening tool. We aimed to elucidate β-hCG expression patterns in CCA tissues and biofluids and to rigorously assess its diagnostic accuracy and prognostic relevance. Furthermore, we evaluated the diagnostic performance of a comprehensive multiparameter serum panel incorporating β-hCG alongside routine cancer biomarkers (AFP, CEA, and CA19-9). This integrated biomarker strategy is designed to significantly enhance sensitivity and specificity of CCA detection, providing a highly translatable and accessible screening method to improve clinical outcomes in regions with a high disease burden.

## 2. Results

### 2.1. Patient Characteristics

The characteristics of the study participants are shown in Table 1. The average age of participants was 62 years. This study included samples from 405 individuals: 153 cases of CCA, 50 cases of HCC, 50 cases of LM, 52 cases of BBD, and 100 healthy controls. Among the 153 CCA cases, 141 had intrahepatic CCA (iCCA) and 12 had extrahepatic CCA (eCCA). The mean age of patients with CCA was 65 years, with similar mean ages (65 and 66 years) observed in the iCCA and eCCA subgroups. The cohort included more males than females (96:57), and this ratio was maintained within the iCCA (89:52) and eCCA (7:5) groups. The papillary histological type was identified in 72 of the CCA cases (68 iCCA and 4 eCCA). Lymph node metastasis was detected in 80 cases, and lymphovascular invasion was detected in 126 cases. Overall, the average tumor volume was 359.38 cm^3^, 365.51 cm^3^ for iCCA, and 287.40 cm^3^ for eCCA. Distant metastasis occurred in 36 patients (34 cases for iCCA and 2 cases for eCCA). Because the eCCA subgroup comprised only 12 cases, these iCCA/eCCA figures are presented for descriptive purposes only. No inferential or subgroup-specific conclusions were drawn from the eCCA data, and all subsequent diagnostic and prognostic analyses refer to the CCA cohort as a whole, which was predominantly intrahepatic.

### 2.2. Multi-Omics Identification of the LHCGR Signaling Axis and Selection of β-hCG

**Figure 1:**
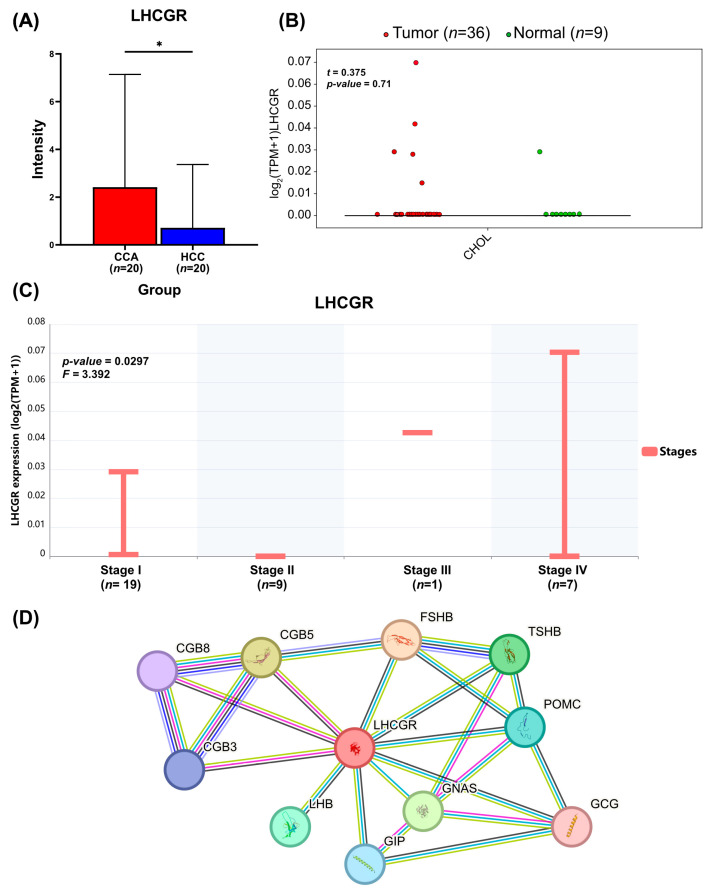
Multi-omics identification and validation of the LHCGR signaling axis as a diagnostic target in cholangiocarcinoma (CCA). (**A**) Serum proteomic profiling demonstrates a significant upregulation of the luteinizing hormone/choriogonadotropin receptor (LHCGR) in the serum of CCA patients (*n* = 20) compared to those with hepatocellular carcinoma (HCC, *n* = 20) (* *p* < 0.05). (**B**) Transcriptomic validation utilizing the GEPIA3 platform, showing LHCGR mRNA expression levels (transcripts per million, TPM) in Chol (CCA) tumor tissues (*n* = 36) (red) compared to normal adjacent tissues (*n* = 9) (green). (**C**) Pathological stage plot (GEPIA3) illustrating a statistically significant correlation between elevated LHCGR transcript expression and clinical tumor stages (Stage I: *n* = 19; Stage II: *n* = 9; Stage III: *n* = 1; Stage IV: *n* = 7) in CCA patients (*p* = 0.0297, F = 3.392). (**D**) Protein–protein interaction network generated via the STRING database. The central LHCGR node demonstrates direct, high-confidence functional interactions with the CGB protein family (including CGB3, CGB5, and CGB8), which comprise the β-subunits of human chorionic gonadotropin (β-hCG), providing the mechanistic rationale for targeting the circulating β-hCG ligand.

To identify novel pathways and potential diagnostic targets for CCA, we initially investigated systemic proteomic alterations in patient serum (unpublished data). Proteomic profiling revealed a significant upregulation of the luteinizing hormone/choriogonadotropin receptor (LHCGR) in the serum of CCA patients (*n* = 20) when compared to those with hepatocellular carcinoma (HCC, *n* = 20) (*p* < 0.05) (Figure 1A). To independently validate this receptor overexpression at the transcriptomic level, we analyzed public RNA-sequencing data from the cholangiocarcinoma (CHOL) cohort using the GEPIA3 platform. The transcriptomic analysis confirmed an increasing trend in LHCGR mRNA expression in CCA tumor tissues compared to normal adjacent tissues (Figure 1B). Notably, the data demonstrated a statistically significant increase in LHCGR expression that correlated with advanced pathological stages (*p* = 0.0297) (Figure 1C). To translate this receptor dysregulation into a viable diagnostic screening strategy, we explored the functional protein–protein interaction network of LHCGR using the STRING database. The network analysis confirmed highly significant biological interactions between LHCGR and the chorionic gonadotropin subunit beta (CGB) protein family (specifically CGB3, CGB5, and CGB8), which encodes the β-subunits of human chorionic gonadotropin (β-hCG) (Figure 1D). The concurrent overexpression of LHCGR in both serum and tissue datasets suggests that CCA tumors may exploit trophoblastic or gonadal signaling pathways to support disease progression. Driven by this multi-omics evidence of an activated LHCGR axis, we hypothesized that the corresponding circulating ligand, ectopic β-hCG, could serve as a highly specific complementary clinical biomarker. Crucially, the selection of β-hCG as the primary target for our clinical validation phase was strongly motivated by diagnostic translatability. While identifying receptor-level (LHCGR) dysregulation provides the biological mechanism, measuring the secreted ligand (β-hCG) in circulation offers a vastly superior clinical approach. β-hCG quantification is a highly standardized, cost-effective, and globally accessible routine automated assay in standard hospital laboratories. By targeting this easily measurable circulating ligand rather than the complex receptor, we ensure that the resulting diagnostic panel can be seamlessly and rapidly implemented into routine hospital-based screening protocols for CCA.

**Figure 2:**
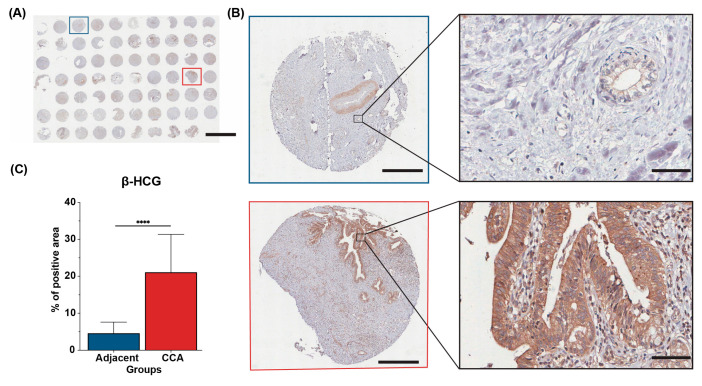
Immunohistochemical analysis of β-hCG expression in cholangiocarcinoma (CCA) and adjacent tissues. (**A**) The tissue array was stained for β-hCG and captured at 1× magnification. The blue box indicates adjacent non-tumor tissue, and the red box indicates CCA tissue (scale = 5 mm). (**B**) The expression levels of β-hCG were assessed in non-tumor adjacent tissues (blue box) and CCA tissues (red box) using immunohistochemical techniques (scale = 50 μm). (**C**) Quantification of β-hCG-positive staining (brown color) intensity in the tissues was quantified using ImageJ software version 1.54k. The histogram represents the mean ± SD of 10 representative areas per sample. Statistical analysis was performed using Student’s *t*-test (**** *p* < 0.0001).

We conducted immunohistochemical staining (IHC) to confirm the relative levels of expression of β-hCG in CCA tissues. TMA analysis revealed a distinct pattern of β-hCG expression in CCA tissues compared with adjacent non-tumor regions (Figure 2A). High-magnification images confirmed weak β-hCG staining in adjacent non-tumor tissues (Figure 2B), whereas intense staining was consistently observed within tumor areas (Figure 2B). Quantitative assessment of β-hCG-positive staining further underscored this difference: CCA tissues exhibited a significantly higher percentage of β-hCG-positive areas than adjacent non-tumor tissues (*p* < 0.0001) (Figure 2C). These findings indicate that β-hCG expression is elevated in CCA, supporting its potential role as a biomarker for this malignancy.

### 2.3. The Prognostic and Diagnostic Significance of Elevated β-hCG Levels in Cholangiocarcinoma

**Figure 3:**
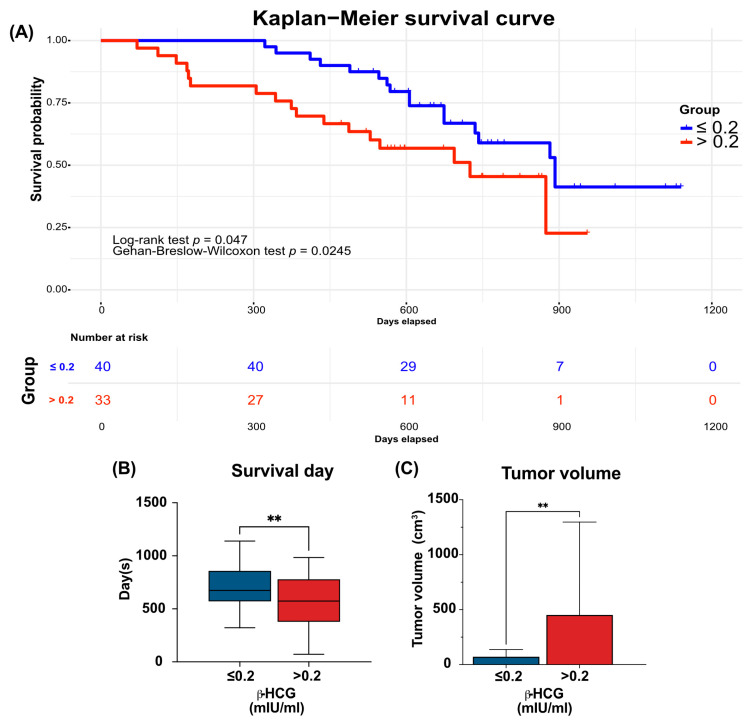
Association of β-hCG levels with tumor volume and survival outcomes in CCA patients. (**A**) Kaplan–Meier survival curves for patients stratified by serum β-hCG levels (≤0.2 vs. >0.2 mIU/mL). The log-rank test (*p* = 0.0467) and Gehan–Breslow–Wilcoxon test (*p* = 0.0245) were used to test for significant differences. (**B**) The average number of survival days of patients with serum β-hCG ≤ 0.2 vs. >0.2 mIU/mL (*p* < 0.01). (**C**) The average tumor volume (cm^3^) in patients classified by serum β-hCG levels (** *p* < 0.01).

Survival analysis demonstrated that patients with detectable β-hCG levels (>0.2 mIU/mL) demonstrated significantly poorer overall survival compared to those with undetectable levels (log-rank *p* = 0.0467). Cox proportional hazards regression confirmed this divergence, yielding a hazard ratio (HR) of 2.086 (95% CI: 1.011–4.308), indicating a substantial and clinically meaningful increase in mortality risk for the detectable group (Figure 3A,B). Patients with detectable β-hCG levels exceeding 0.2 mIU/mL also exhibited significantly larger tumor volumes (*p* < 0.05; Figure 3C), suggesting a strong correlation between any measurable ectopic β-hCG expression and accelerated tumor progression.

### 2.4. Detection of Tumor Biomarkers in Serum

**Figure 4:**
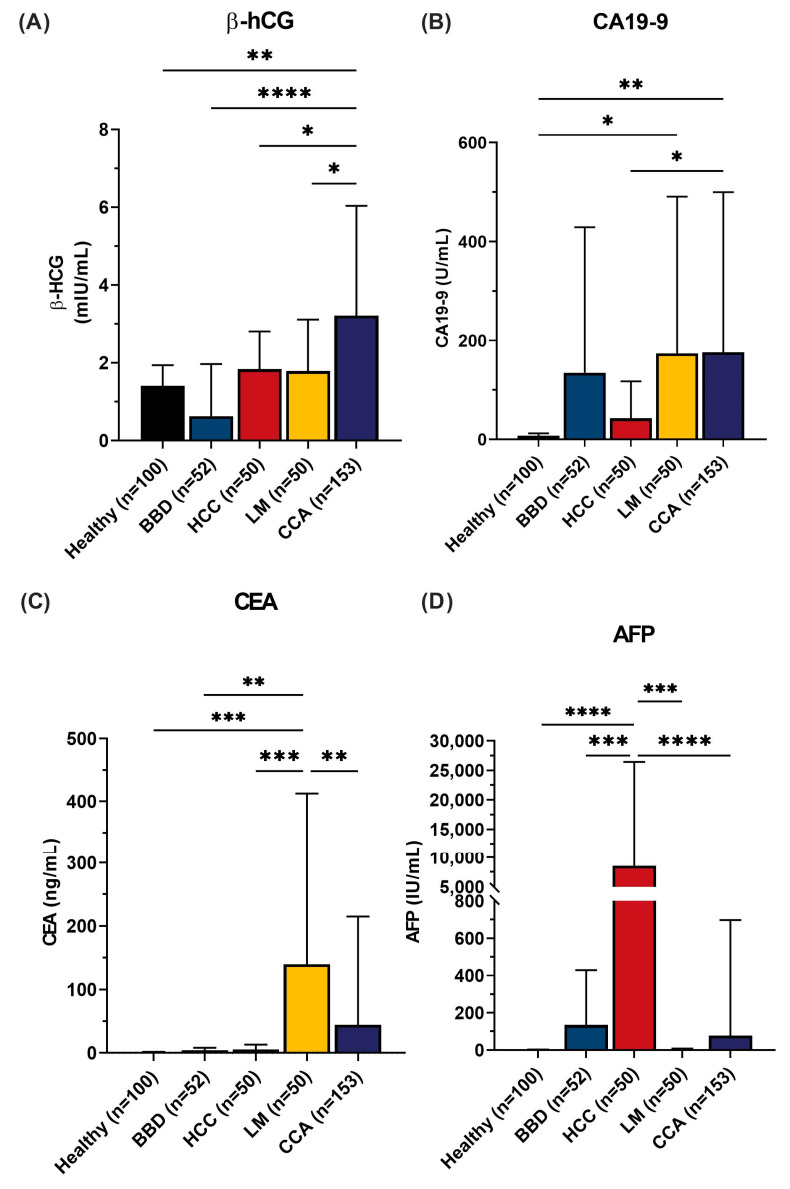
Comparison of serum glycobiomarker levels among healthy controls and hepatocellular carcinoma (HCC), liver metastases (LM), and cholangiocarcinoma (CCA) cases. (**A**) Serum β-hCG levels were significantly elevated in all cancer groups (HCC, LM, CCA) compared to healthy controls, with the highest levels observed in the CCA group. (**B**) Serum CA19-9 levels were markedly higher in the CCA and BBD groups compared to LM, HCC, and healthy controls, with statistically significant differences across groups. (**C**) CEA levels were significantly higher in the LM and CCA groups. (**D**) AFP levels were dramatically elevated only in the HCC group compared to LM, CCA, and healthy controls (* *p* < 0.05, ** *p* < 0.01, *** *p* < 0.001, **** *p* < 0.0001).

We measured serum tumor biomarkers in patients with hepatocellular carcinoma (HCC), liver metastases (LM), benign biliary disease (BBD) and CCA and compared them with those in healthy controls. We found that β-hCG levels were significantly higher in CCA, followed by LM and HCC, indicating the potential of β-hCG as a diagnostic marker (Figure 4A). Carbohydrate antigen 19-9 (CA19-9) levels showed significant elevations, particularly in the CCA group (Figure 4B). Carcinoembryonic antigen (CEA) levels were markedly higher in LM patients (Figure 4C). Alpha-fetoprotein (AFP) levels were dramatically higher in HCC patients, with significant decreases also observed in LM and CCA groups (Figure 4D).

### 2.5. The Diagnostic Performance of the Biomarkers

**Figure 5:**
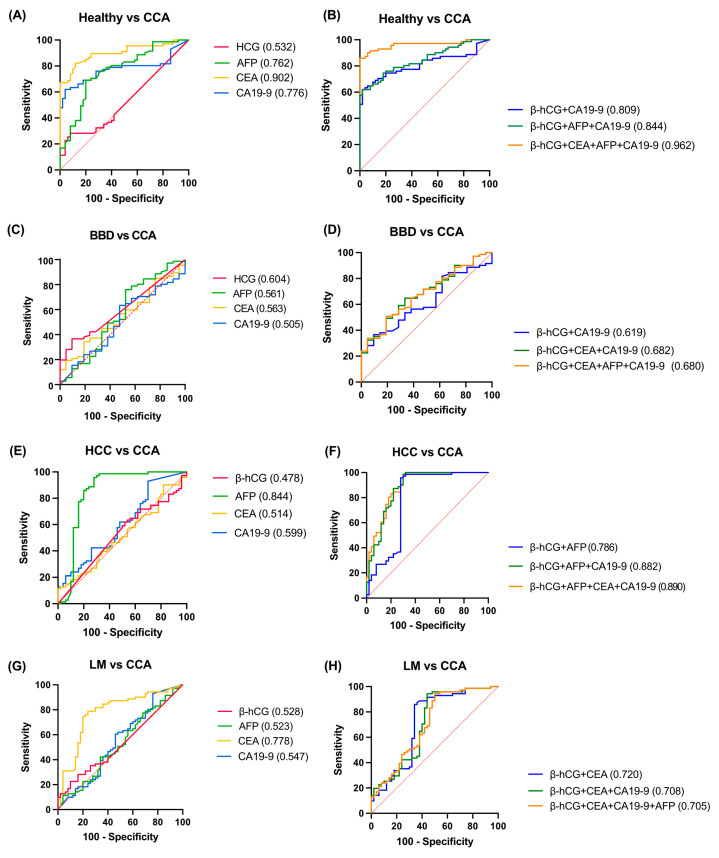
Receiver operating characteristic (ROC) curves for biomarkers in differentiating various health conditions from cholangiocarcinoma (CCA). (**A**,**B**) ROC curves comparing healthy controls to CCA cases. (**C**,**D**) ROC curves comparing benign biliary disease (BBD) to CCA. (**E**,**F**) ROC curves comparing hepatocellular carcinoma (HCC) to CCA. (**G**,**H**) ROC curves comparing liver metastases (LM) to CCA.

The diagnostic performance of β-hCG alone and in combination with other biomarkers (CA19-9, AFP, CEA) was evaluated across different patient groups (Figure 5 and Table 2). When distinguishing CCA from healthy controls, β-hCG alone showed limited diagnostic value (AUC = 0.532; sensitivity 28%; specificity 92%). However, combining β-hCG with CA19-9, AFP, and CEA markedly improved diagnostic performance, achieving the highest AUC (0.962), sensitivity (86%), and near-perfect specificity among all comparisons. It is important to underscore that this highly elevated classification metric may partially reflect model overfitting, given the lack of an independent internal or external validation dataset and the limited sample sizes inherent to specific rare pathological presentation subgroups, such as eCCA (*n* = 12). When comparing CCA with BBD, β-hCG alone again demonstrated limited performance (AUC = 0.604; sensitivity 37%, specificity 91%). The multi-marker panel (β-hCG, CA19-9, AFP, CEA) moderately enhanced diagnostic accuracy for BBD (AUC = 0.680; sensitivity 51%, specificity 81%). In differentiating CCA from HCC, β-hCG alone performed poorly (AUC = 0.478; sensitivity 13%, specificity 96%). Notably, incorporating AFP, CA19-9, and CEA significantly improved the ability to distinguish between HCC and CCA. The combined biomarker panel, which also included β-hCG, reached high diagnostic efficacy with an AUC of 0.890, a sensitivity of 100%, and a specificity of 68%. For the comparison of CCA with LM, β-hCG alone showed minimal diagnostic utility (AUC = 0.528; sensitivity 23%, specificity 90%). However, the combined biomarker panel substantially improved discrimination between these conditions (AUC = 0.705; sensitivity 94%, specificity 50%). Collectively, these results indicate that while β-hCG alone has limited standalone diagnostic capability, its combination with established tumor markers significantly enhances classification trends across all patient groups, supporting its potential inclusion in an exploratory multi-marker diagnostic panel for CCA detection.

## 3. Discussion

The principal finding of this study is the significant enhancement of diagnostic accuracy for cholangiocarcinoma (CCA) by integrating β-hCG into a standard biomarker panel consisting of AFP, CEA, and CA19-9. This discovery addresses a critical clinical need in resource-limited regions burdened by *Opisthorchis*-associated CCA, such as Northeast Thailand, where the high cost and inaccessibility of advanced imaging modalities like CT and MRI frequently lead to delayed diagnoses and poor prognoses. Our results demonstrate that a relatively inexpensive, serum-based biomarker panel incorporating β-hCG offers a practical and powerful screening tool that can be readily implemented in primary care settings for screening at-risk individuals in these high-prevalence areas.

Our investigation to identify novel systemic markers began with a multi-omics bioinformatics approach rather than traditional direct ligand screening. Recognizing that CCA tumor cells manipulate their microenvironment through complex cell-surface signaling, our initial serum proteomic screening specifically compared CCA with hepatocellular carcinoma (HCC). This direct comparison was strategically chosen because HCC is the most prevalent primary liver cancer and frequently presents a major differential diagnostic challenge from intrahepatic CCA [19]. From this comparative analysis, we identified significant upregulation of the luteinizing hormone/choriogonadotropin receptor (LHCGR) in CCA patients, a finding we further validated using public transcriptomic datasets. This observation aligns with existing evidence demonstrating that aberrant expression of LHCGR frequently occurs in various non-trophoblastic malignancies, where it facilitates autocrine or paracrine signaling to promote aggressive tumor behaviors [20,21]. Because targeting complex cell-surface receptors is clinically impractical for routine, high-throughput hospital screening, we utilized protein–protein interaction network analysis (STRING) to map the receptor’s corresponding ligands. This network confirmed a direct, high-confidence biological linkage between the dysregulated LHCGR axis and the chorionic gonadotropin subunit beta (CGB) protein family, which comprises the β-subunit of human chorionic gonadotropin (β-hCG) [22]. Building upon prior literature that has documented elevated β-hCG expression in gastrointestinal cancers and pre-malignant biliary conditions such as primary sclerosing cholangitis [14,15,16,17,18], we strategically selected circulating β-hCG as our primary target for downstream clinical validation. The rationale for this transition is grounded in both biological precedent and strict clinical pragmatism: while the receptor provides the biological mechanism of disease, the secreted ligand (β-hCG) is commonly and inexpensively measured in routine automated clinical laboratories, maximizing its diagnostic translatability [23].

The value of β-hCG as a tumor marker has been shown in several cancers such as ovarian cancer [24], germ-cell tumors [25], and head and neck cancers [11]. Elevated serum β-hCG levels are observed in 45–60% of patients with biliary and pancreatic cancers [17]. It is possible that hCG is produced by liver tumors [14]. We confirmed our systems biology hypothesis by staining for β-hCG in a CCA tissue array, demonstrating significantly higher β-hCG immunoreactivity directly within tumor areas compared to adjacent non-tumor tissues, validating its origin from malignant cells. The ectopic expression of β-hCG in these tissues [26] points to a possible function beyond its traditional role in pregnancy. It is highly probable that the β-hCG subunit, through interaction with the upregulated LHCGR axis we identified, contributes to tumor proliferation and survival by activating autocrine or paracrine signaling pathways that support cell growth and inhibit apoptosis [27]. Furthermore, we found that higher detectable serum β-hCG levels in CCA patients correlated with a worse prognosis. This finding is consistent with previous studies showing that hCG-positive colon [16] and prostate [28] cancers also have poorer outcomes than their hCG-negative counterparts. Most of our CCA cohort had been diagnosed with intrahepatic CCA (iCCA) reflecting the rising incidence of this form globally [29] as well as in Thailand [30]. In regions where *O. viverrini* (OV) infection is endemic, particularly Northeast Thailand [2], iCCA is considerably more common than extrahepatic CCA (eCCA), largely attributed to chronic inflammation and bile duct damage caused by OV infection [2]. The gender ratio of our cohort also aligns with the generally higher prevalence of CCA among males [31].

Serum β-hCG is a promising biomarker for CCA, as its levels are significantly elevated in CCA patients compared to those with benign biliary diseases or other liver cancers. β-hCG levels were significantly elevated in CCA patients compared to those with hepatocellular carcinoma (HCC), liver metastases (LM), benign biliary disease (BBD), and healthy controls. The highest levels of β-hCG were observed in CCA patients. This marker appears to be produced directly by tumor cells and secreted into the bile, and its presence in high-risk, non-cancerous conditions like primary sclerosing cholangitis [18] suggests it could be a valuable tool for early screening. However, its performance as a standalone diagnostic test is only moderate, with a sensitivity of around 51% and specificity of 81%, due to the occurrence of false positives in benign conditions and false negatives in non-producing tumors. Consequently, while extremely high levels strongly indicate cancer, there is an ambiguous ‘overlap zone’ where other diagnostic tools like imaging are necessary for confirmation. Beyond its integration into the complete four-biomarker panel, the inclusion of β-hCG provides a distinct, quantifiable improvement in the diagnostic performance of CA19-9 alone. In routine practice, CA19-9 levels are frequently elevated due to benign biliary inflammation or cholestasis, limiting its standalone reliability. Our data demonstrate that pairing β-hCG directly with CA19-9 as a dual-marker baseline successfully increases the area under the curve (AUC) from 0.776 to 0.809 against healthy controls. More notably, when differentiating CCA from benign biliary diseases, the dual-marker panel improved the AUC from a baseline of 0.505 up to 0.619 (Table 2). This specific performance gain indicates that ectopic β-hCG secretion acts as an independent serological indicator of malignancy, directly improving comprehensive risk recognition by filtering out false-negative cases that standard CA19-9 tracking fails to identify.

While traditional markers such as CEA demonstrated a strong standalone diagnostic capacity within our cohort (AUC = 0.902), evaluating performance solely against healthy controls oversimplifies the real-world clinical challenge of diagnosing CCA. The most significant diagnostic hurdle is differentiating CCA from confounding conditions such as BBD and other liver malignancies, where traditional glycoprotein markers frequently yield false positives due to severe inflammation or cholestasis. For instance, standalone CEA achieves a specificity of only 88% when differentiating CCA from BBD. The integration of β-hCG into the multiparameter panel is intentionally designed to address this critical specificity deficit. Ectopic β-hCG secretion operates through an orthogonal biological pathway, acting as a highly specific indicator of tumor dedifferentiation rather than generalized biliary stress. Consequently, despite β-hCG exhibiting a modest standalone AUC, its complementary value functions as a definitive diagnostic safety net, filtering out the false-positive benign cases that CEA fails to distinguish. By combining these independent molecular axes, the comprehensive panel successfully elevates the overall diagnostic AUC to 0.962. This integration achieves optimal specificity within our evaluated cohort while maintaining a sensitivity of 86%, representing a vital quantitative reduction in clinical misclassification compared to relying on any single traditional marker.

The diagnostic utility of β-hCG is markedly improved when it is included in a panel with AFP, CA19-9, and CEA. This combined panel effectively distinguishes CCA from healthy controls with high accuracy (AUC 0.96; 86% sensitivity, near-perfect specificity). The synergy of this multi-marker strategy comes from each biomarker providing independent and complementary clinical information: AFP helps separate CCA from HCC, while CEA and CA19-9 help distinguish it from liver metastases. AFP is typically elevated in HCC but remains low in CCA [32]. Likewise, CEA and CA19-9 help distinguish primary biliary malignancies from metastatic liver lesions originating in organs like the colon or pancreas, which can otherwise present with overlapping clinical features. In this study, we specifically focused on liver metastases originating from colorectal cancer, as it frequently metastasizes to the hepatic parenchyma [33]. By leveraging the combined strengths of these markers alongside the specific elevation of β-hCG in CCA, clinicians can achieve a more comprehensive and reliable profile than would be possible using any single marker in isolation. However, as a critical caveat for real clinical practice, the discriminatory performance of this panel declines significantly when faced with confounding hepatic conditions rather than healthy controls. Specifically, in distinguishing CCA from BBD, the four-biomarker panel yielded a moderate AUC of 0.680, alongside a relatively low sensitivity of 51%. More concerningly, when distinguishing CCA from LM, the panel yielded an AUC of 0.705 and an unacceptably low specificity of 50%. In a true diagnostic screening pipeline, a 50% specificity against metastatic disease would result in a prohibitive rate of false-positive results, placing unnecessary psychological and financial burdens on patients. Therefore, these data must be interpreted cautiously, and the panel should not be misconstrued as a definitive, standalone tool for ‘early detection’ or universal screening. Instead, its true clinical utility may lie in its exceptionally high sensitivity (94%) when cross-referenced against LM. This high sensitivity allows the panel to function effectively as an affordable, first-line serological triage mechanism in high-incidence, resource-constrained regions. Rather than providing an absolute, independent diagnosis, an elevated panel score would serve as an early red flag, effectively stratifying high-risk individuals who require immediate, prioritized referral for gold-standard imaging modalities such as magnetic resonance cholangiopancreatography (MRCP), contrast-enhanced CT, or definitive histopathological verification.

It is worth noting that our prognostic threshold of 0.2 mIU/mL represents the literal lower limit of detection (LoD) of the automated clinical analyzer utilized. While the normal reference range for healthy, non-pregnant cohorts is broadly capped at <5 mIU/mL in macro-diagnostic settings, our findings imply that within an oncological context, the transition from completely undetectable (≤0.2 mIU/mL) to detectable (>0.2 mIU/mL) trace serum concentrations carries distinct prognostic weight. This indicates that even minute, ectopic leakage of β-hCG into the circulation, though well below standard pregnancy thresholds serves as a highly sensitive indicator of increased tumor volume and aggressive clinical progression. For real-world hospital translation, utilizing the automated platform’s baseline LoD as a binary indicator (detectable vs. undetectable) provides a highly reproducible strategy for patient risk-stratification without requiring customized clinical assay setups. For β-hCG quantification using the Elecsys HCG+β assay, the manufacturer-defined analytical limits are as follows: the limit of blank is 0.100 mIU/mL, the LoD is 0.200 mIU/mL, and the limit of quantitation (LoQ) is 0.600 mIU/mL. The LoQ is defined as the lowest analyte concentration that can be reproducibly measured with an intermediate precision coefficient of variation (CV) of ≤20%. Because the prognostic threshold of 0.2 mIU/mL utilized in our survival analysis aligns directly with the assay’s baseline LoD rather than the LoQ, this stratification functionally serves as a binary indicator. It compares patients with completely undetectable β-hCG levels (≤0.2 mIU/mL) against those exhibiting any detectable trace of ectopic marker expression (>0.2 mIU/mL), thereby mitigating concerns regarding quantitative variance at the extreme lower limits of the analytical spectrum.

The fundamental methodological and statistical limitations of the present study must be addressed. First, the multiparameter diagnostic panel was both derived and evaluated on the identical patient dataset, without the benefit of a separate, independent validation cohort. This circular validation design means the highly favorable AUC values particularly against healthy controls are susceptible to overfitting and must be viewed as an exploratory baseline rather than a clinically cross-validated metric. Second, while the total cohort is large (*n* = 405), certain crucial subcategories remained highly restricted, most notably the extrahepatic CCA (eCCA) cohort, which consisted of only 12 cases. This subgroup is too small to support generalizable inference; accordingly, we refrain from drawing any eCCA-specific conclusions, and our findings should be interpreted as applying primarily to the intrahepatic-dominant CCA cohort. Furthermore, we acknowledge that while our multi-omics bioinformatics discovery phase provides a strong biological rationale for selecting β-hCG, this in silico pathway analysis requires further mechanistic validation. However, we immediately translated this target into large, independent cohorts, validating β-hCG expression across 100 tissue microarray specimens and standardizing its detection in the serum of 405 individuals. This extensive downstream validation strongly supports β-hCG as a clinically justified candidate. These combined constraints are a direct consequence of the pilot nature of this translational study, reinforcing that these findings represent an essential steppingstone that strictly requires external, multicenter clinical validation before real-world implementation.

## 4. Materials and Methods

The study protocol was based on the Declaration of Helsinki and the ICH good clinical practice guidelines. This study was approved by the Khon Kaen University Human Research Ethics Committee under reference number HE 671 702 for serum and HE 591 298 for tissue arrays.

### 4.1. Serum Samples from Patients with Different Liver Diseases and Controls

Serum samples (*n* = 405) were from 153 cases of cholangiocarcinoma (CCA), 50 cases of hepatocellular carcinoma (HCC), 50 cases of liver metastases (LM), 52 cases of clinically diagnosed benign biliary disease (BBD), and 100 healthy controls. The BBD group comprised patients with conditions such as gallstones, primary sclerosing cholangitis, and other cholestatic liver diseases. All samples were leftover samples that had been collected at Srinagarind Hospital, Faculty of Medicine, Khon Kaen University. Briefly, blood samples were obtained by venipuncture into serum separation tubes (SST). After allowing clotting, the samples were centrifuged at 3500 rpm for 10 min to separate the serum. Following completion of routine laboratory procedures, all serum samples were stored at −80 °C until biomarker measurement or proteomics analysis.

### 4.2. Liquid Chromatography–Tandem Mass Spectrometry (LC/MS–MS) Analysis

The protein concentration of each sample was measured with the Lowry assay using bovine serum albumin (BSA) as the standard protein. Disulfide bonds in the protein samples were reduced by incubating them with 10 mM dithiothreitol in 10 mM ammonium bicarbonate. The samples were then incubated with 100 mM iodoacetamide in 10 mM ammonium bicarbonate in the dark for 1 h at room temperature to alkylate cysteine residues in the proteins. The protein samples were digested overnight at room temperature with trypsin (Promega, Walldorf, Germany) at a ratio of 1:20 (*w/w*). Enzymatic digestion was stopped using 0.1% formic acid. The peptide samples were analyzed using a liquid chromatography system (Thermo Scientific, Waltham, MA, USA) connected to a hybrid quadrupole Q-Tof Impact II (Bruker, Billerica, MA, USA) with a nano captive-spray ion source. Peptide digests were packed with Acclaim PepMap RSLC C18, 2 μm, 100 Å, and nanoViper after being enriched on a pre-column 300 μm i.d. × 5 mm C18 Pepmap 100, 5 μm, 100 Å (Thermo Scientific). The C18 column was surrounded by a thermostated column oven set at 60 °C. On the analytical column, solvents A (containing 0.1% formic acid in water) and B (containing 0.1% formic acid in 80% acetonitrile) were provided.

Peptides were eluted for 30 min at a constant flow rate of 300 nL/min with a gradient of 5–55% solvent B. CaptiveSpray was used for 1.6 kV electrospray ionization. Approximately 50 L/h of nitrogen was employed as the drying gas. Collision-induced-dissociation (CID) product ion mass spectra were obtained using nitrogen gas as the collision gas. Mass spectra in positive ion mode and MS/MS spectra were obtained at 2 Hz over the range (*m/z*) 150–2200. Based on the *m/z* value, the collision energy was changed to 10 eV. The LC-MS analysis was performed three times with each sample.

DeCyder MS Differential Analysis software version 6.5 (DeCyderMS, GE Healthcare Life Science, UK) was used to quantify and identify the proteome of each tissue type. The analyzed MS/MS data from DeCyderMS were submitted for a database search using the Mascot software version 3.1 (Matrix Science, London, UK). The data were searched against the NCBI database for protein identification. The database search parameters were configured as follows: taxonomy (*Homo sapiens*); enzyme (trypsin); variable modifications (carbamidomethyl, oxidation of methionine residues, Hex(3)HexNAc(1)Pent(1)(N), Hex(3)HexNAc(2)(N), Hex(3)HexNAc(2)-P(1)(N), Hex(3)HexNAc(4)(N), Hex(4)HexNAc(4)(N), Hex(5)HexNAc(2)(N), Hex(5)HexNAc(4)(N), Hex1HexNAc1(S), Hex1HexNAc1(T)); mass values (monoisotopic); protein mass (unrestricted); peptide mass tolerance (1.2 Da); fragment mass tolerance (±0.6 Da); peptide charge states (1+, 2+, and 3+); and a maximum of 2 missed cleavages. Identified proteins were filtered using one-way ANOVA (*p* < 0.05). In this experiment, 5 μg of BSA was used as an internal standard to normalize the protein intensities from each run.

### 4.3. Bioinformatics Discovery Phase

To establish the biological rationale for biomarker selection, a multi-tiered discovery approach was employed. First, relative quantification of the luteinizing hormone/choriogonadotropin receptor (LHCGR) was extracted from our internal serum proteomic profiles comparing CCA and HCC patients. To validate these findings at the transcriptomic level, public RNA-sequencing data from the cholangiocarcinoma cohort (TCGA-CHOL) were analyzed using the Gene Expression Profiling Interactive Analysis (GEPIA3) web server (https://gepia3.bioinfoliu.com (accessed on 8 June 2026)). Differential mRNA expression of LHCGR was evaluated between CCA tumor tissues and normal adjacent tissues. Furthermore, pathological stage analysis (Stages I–IV) was conducted within GEPIA3 to assess the correlation between LHCGR transcript levels and disease progression, with statistical significance determined by one-way ANOVA. Finally, a functional protein–protein interaction (PPI) network centered on LHCGR was constructed using the STRING database version 12.0 [34]. The network was generated utilizing the default active interaction sources (including text mining, experiments, databases, and co-expression), with the minimum required interaction score strictly set to high confidence (0.700). This interaction analysis was performed to map direct biological linkages between the upregulated receptor and potential circulating ligands, specifically identifying the *CGB* gene family encoding β-hCG.

### 4.4. Quantification of Serum Tumor Markers

Levels of serum beta-human chorionic gonadotropin (β-hCG), alpha-fetoprotein (AFP), carbohydrate antigen 19-9 (CA19-9) and carcinoembryonic antigen (CEA) were determined using an automated analyzer (Elecsys e801, cobas 8000 modular analyzer series, Roche Diagnostics, Mannheim, Germany) at the clinical laboratory section, Srinagarind Hospital, Faculty of Medicine, Khon Kaen University. The internal (PC TM, Roche, Mannheim, Germany) and external (Riqas, Randox Laboratories, Antrim, UK) standard quality-control materials were used for evaluating the instrument’s precision and accuracy. The lower limit of detection for the automated serum β-hCG assay on this platform was defined by the manufacturer as 0.2 mIU/mL.

### 4.5. Immunohistochemical Study

Tissue microarrays (TMAs) were constructed utilizing 100 histologically confirmed CCA samples and their corresponding paired adjacent non-tumor tissues (*n* = 100). Additionally, the arrays included external anatomical control cores (two normal colon, two gallbladder, and two pancreas) to confirm the biliary origin of the tissues. These were included strictly to serve as external anatomical reference controls for antibody validation and staining consistency; consequently, these supplementary tissues were excluded from all comparative biological analyses. All tissues utilized for TMA construction were obtained from archival residual surgical specimens provided by the Department of Pathology, Faculty of Medicine, Khon Kaen University.

Briefly, TMA specimens were sectioned to a thickness of 5 µm, deparaffinized in xylene, and rehydrated through a descending ethanol series. For antigen retrieval, the sections were autoclaved in a citrate buffer solution. For immunostaining, slides were incubated overnight at 4 °C with a primary rabbit polyclonal anti-β-hCG antibody (1:100 dilution, ab53087, Lot No. GR88653-47; Abcam, Cambridge, MA, USA). Subsequently, the sections were incubated with a goat anti-rabbit horseradish peroxidase (HRP)-conjugated secondary antibody (111-035-003; Jackson ImmunoResearch, West Grove, PA, USA). Immunoreactivity was visualized via the addition of a 3,3′-diaminobenzidine (DAB) chromogen solution (Merck, Darmstadt, Germany). The slides were then counterstained with Mayer’s hematoxylin for 2 min, rinsed with distilled water, and dehydrated using ethanol and xylene. To assess protein expression, ten representative, randomly selected fields of view from each slide were examined under light microscopy. Rather than relying on subjective manual grading, the relative immunohistochemical staining intensity was digitally evaluated and objectively quantified utilizing ImageJ software (National Institutes of Health, Bethesda, MD, USA), as previously described for the semi-quantitative determination of protein expression [35].

### 4.6. Statistical Analysis

Data obtained from at least three independent samples are presented as mean ± SD. For comparisons involving two groups, the Student’s *t*-test or chi-square test was used. When analyzing multiple groups, a one-way ANOVA or Kruskal–Wallis test was performed, followed by Tukey’s post hoc test or Dunn’s multiple comparison test as appropriate. To rigorously control the Type I error rate inflation associated with multiple testing across the numerous clinical cohorts and various biomarkers, subsequent pairwise post hoc analyses were conducted using Dunn’s test with Bonferroni correction applied to the *p*-values. Kaplan–Meier survival analysis with the log-rank test was conducted to compare survival distributions between low and high β-hCG levels in serum samples from patients with CCA. Receiver operating characteristic (ROC) analysis was used to determine the optimal cut-off for maximum sensitivity and specificity. Statistical analyses were performed using GraphPad Prism version 10.2.1 (GraphPad Software, MA, USA) for macOS 14 and SPSS version 29 (SPSS Inc., IL, USA). Statistical significance was defined as *p* < 0.05.

## 5. Conclusions

Our findings establish that while β-hCG serves as a promising biomarker for CCA, the primary strength of the integrated multi-marker panel (β-hCG, AFP, CEA, and CA19-9) lies specifically in its exceptional accuracy for distinguishing CCA patients from healthy individuals. However, the panel’s discriminatory performance decreases significantly when evaluated against other confounding hepatobiliary pathologies, such as benign biliary diseases and liver metastases, which restricts its current utility as a definitive, universal standalone screening test. Consequently, further extensive clarifications, including the optimization of biomarker cut-off thresholds and the exploration of additional highly specific target molecules, are critically needed to improve the panel’s efficacy in true differential diagnosis. Future research must focus on validating and refining these findings in large-scale, prospective, multicenter cohorts to safely bridge the gap between initial serological stratification and definitive clinical translation.

## Figures and Tables

**Table 1:** Baseline characteristics of the study participants.

Characteristics	Participants
Total (*n*)	405
Age (Mean ± 2SD)	62 ± 18
Male:Female	225:180
Healthy	100
BBD	52
CCA	153
HCC	50
Liver metastases	50

CCA, Cholangiocarcinoma; BBD, Benign biliary diseases; HCC, Hepatocellular carcinoma.

**Table 2:** Diagnostic performance of biomarkers for CCA diagnosis.

Group Comparison	Glyco-Biomarkers	AUC	95% CI	*p*-Values	Cut Off Values	Youden Index	Sensitivity (%)	Specificity (%)	PPV (%)	NPV (%)	Accuracy (%)
CCA vs. Healthy	β-hCG	0.532	0.429–0.635	0.554	1.95	0.21	28	92	83	47	55
	CA19-9	0.776	0.691–0.861	<0.0001	16.58	0.58	62	96	96	64	76
	AFP	0.762	0.675–0.849	<0.0001	2.25	0.48	68	80	83	66	74
	CEA	0.902	0.854–0.96	<0.0001	2.09	0.71	83	88	91	79	85
	β-hCG, CA19-9	0.809	0.731–0.888	<0.0001	0.63	0.59	63	96	96	65	77
	β-hCG, AFP, CA19-9	0.844	0.777–0.912	<0.0001	0.72	0.59	61	98	98	64	77
	β-hCG, CEA, AFP, CA19-9	0.962	0.927–0.996	<0.0001	0.73	0.86	86	100	100	82	91
CCA vs. BBD	β-hCG	0.604	0.481–0.728	0.1480	0.76	0.28	37	91	93	29	48
	CA19-9	0.505	0.366–0.644	0.944	14.73	0.16	21	95	82	30	61
	AFP	0.561	0.408–0.715	0.397	2.09	0.24	76	48	83	37	70
	CEA	0.563	0.433–0.693	0.382	8.62	0.16	21	95	93	26	38
	β-hCG, CA19-9	0.619	0.496–0.742	0.099	0.77	0.28	37	91	93	30	49
	β-hCG, CEA, CA19-9	0.682	0.562–0.802	0.012	0.70	0.32	65	67	84	35	66
	β-hCG, CEA, AFP, CA19-9	0.680	0.559–0.799	0.013	0.72	0.32	51	81	90	33	58
CCA vs. HCC	β-hCG	0.478	0.374–0.582	0.683	4.21	0.09	13	96	82	44	47
	CA19-9	0.599	0.496–0.702	0.064	0.61	0.23	93	30	65	75	67
	AFP	0.844	0.756–0.932	<0.0001	5.35	0.66	85	80	86	80	83
	CEA	0.514	0.41–0.618	0.788	51.35	0.11	11	100	100	44	48
	β-hCG, AFP	0.786	0.689–0.882	<0.0001	0.73	0.68	96	72	83	95	87
	β-hCG, AFP, CA19-9	0.882	0.816–0.948	<0.0001	0.65	0.69	99	70	82	97	87
	β-hCG, CEA, AFP, CA19-9	0.890	0.829–0.952	<0.0001	0.61	0.68	100	68	82	100	87
CCA vs. LM	β-hCG	0.528	0.425–0.632	0.597	2.96	0.13	23	90	76	45	50
	CA19-9	0.547	0.439–0.654	0.381	0.63	0.16	92	24	62	68	64
	AFP	0.523	0.417–0.629	0.666	1.99	0.09	79	30	61	47	57
	CEA	0.778	0.691–0.866	<0.0001	8.205	0.54	78	76	82	71	77
	β-hCG, CEA	0.720	0.62–0.821	<0.0001	0.58	0.53	89	64	75	85	78
	β-hCG, CEA, CA19-9	0.708	0.608–0.808	<0.0001	0.57	0.50	94	56	73	66	70
	β-hCG, CEA, AFP, CA19-9	0.705	0.607–0.802	<0.0001	0.56	0.44	94	50	73	81	74

CCA, Cholangiocarcinoma; BBD, Benign biliary diseases; HCC, Hepatocellular carcinoma; LM, Liver metastases; AUC, Area under the curve; PPV, Positive predictive value; NPV, Negative predictive value. The 100% specificity represents the exact mathematical output generated from this specific, limited study cohort. It is not intended to represent an absolute clinical guarantee of zero false positives in broader, unselected populations. Thresholds for individual biomarkers are presented in standard clinical units (e.g., mIU/mL, U/mL). Thresholds for combined biomarker panels represent unitless predicted probabilities generated via binary logistic regression equations. The optimal thresholds were determined by calculating the maximum Youden index.

## Data Availability

The data sets used and analyzed in this study are available from the corresponding author on reasonable request.

## Abbreviations

The following abbreviations are used in this manuscript:

β-hCG	Beta-human chorionic gonadotropin
CCA	Cholangiocarcinoma
LM	Liver metastases
BBD	Benign biliary disease
HCC	Hepatocellular carcinoma
AFP	Alpha-fetoprotein
CA19-9	Carbohydrate antigen 19-9
CEA	Carcinoembryonic antigen
LHCGR	Luteinizing hormone/choriogonadotropin receptor
